# 2061: Prevalence, associated characteristics, and diagnostic and treatment process experiences of women seeking emergency department care after being strangled: A mixed methods study

**DOI:** 10.1017/cts.2017.242

**Published:** 2018-05-10

**Authors:** Michelle Patch, Jacquelyn Campbell

## Abstract

OBJECTIVES/SPECIFIC AIMS: Aim 1—estimate prevalence and associated characteristics of nonfatal, non-self-inflicted strangulation among women ages 18 and older who presented to a US emergency department between 2006 and 2013. Aim 2—explore care-seeking behaviors, the context of the care seeking, treatment expectations and perceived diagnosis in a sample of women ages 18 and older who present to a US emergency department and report being strangled by an intimate partner. Aim 3—merge and synthesize findings from both the quantitative and qualitative strands to provide a more complete understanding of post-strangulation emergency care of women. METHODS/STUDY POPULATION: This mixed-methods study will use a convergent parallel design, with a single phase of concurrent and independent data collection. Analysis of quantitative and qualitative data will be performed separately then compared, with main findings integrated during the interpretation phase and presented in a merged data analysis display. IRB review and approval will be obtained before initiating this study. Aim 1 will include a cross-sectional analysis of 2006–2013 Nationwide Emergency Department Sample (NEDS) data, from the Agency for Healthcare Research and Quality’s Healthcare Cost and Utilization Project (HCUP). NEDS is the US’s largest all-payer emergency department (ED) database, providing national estimates of hospital-based ED visits from ~120 to 135 million ED visits/year (weighted). For this study, we will examine data from patients meeting inclusion criteria with an International Classification of Diseases, 9th Revision, Clinical Modification (ICD-9-CM; Medicode, 1996) code of strangulation. For this strand, females aged 18 years or older who presented to a US emergency department between 2006 and 2013 will be included. The outcome variable will be non-fatal, non-self-inflicted strangulation, defined using at least one of the ICD-9-CM codes for strangulation. These codes are: 994.7 (“asphyxiation and strangulation”), E963 (“assault by hanging and strangulation”), E983.8 (“strangulation or suffocation by other specified means undetermined whether accidentally or purposely inflicted”), and E983.9 (“strangulation or suffocation by unspecified means undetermined whether accidentally or purposely inflicted”). Patients with a concurrent ICD-9-CM code for suicide attempt (E953, “Suicide and self-inflicted injury by hanging, strangulation and suffocation”) will be excluded, to minimize self-inflicted assault events. Aim 2 will employ a narrative descriptive approach, with semistructured individual interviews to gather more information about women’s experiences when engaging the health care system after being strangled. Medical records related to the strangulation event will also be reviewed for diagnostic codes and other nursing and/or medical notes that may relate to diagnoses, treatment and referrals. For this strand, women aged 18 years or older who present for care to an urban, academic ED will be recruited, purposely sampling those reporting strangulation as a reason for their visit. We anticipate interviewing ~20–30 women to achieve saturation of information. RESULTS/ANTICIPATED RESULTS: Data from the NEDS from 2006 to 2013 will be analyzed for prevalence and associated characteristics of women seeking care after being strangled. Individual interviews and medical record reviews of a small sample of adult women will be conducted to explore women’s in-depth experiences within the health care system. Results from both the quantitative and qualitative analyses will then be collectively compared and interpreted to better synthesize the evidence from this work. Convergent and divergent findings will be presented in a merged data analysis display (Creswell and Plano Clark, 2011). Qualitative data will be used to fill the knowledge gap remaining from the quantitative analysis, and to explain and contextualize some of the findings. Such integration will help expand the current limited evidence on care of strangled women, and will identify additional research questions that will guide future research in this area. DISCUSSION/SIGNIFICANCE OF IMPACT: To our knowledge, this study will be the first to explore this issue using a nationally representative sample of adult women who sought emergency medical care for strangulation analyzed in conjunction with a detailed qualitative analysis of strangled women’s experiences with the health care system. The resulting knowledge will be critical to informing clinical assessment, intervention and prevention efforts for this vulnerable population, as well as public policy and future research regarding this specific violence tactic.

